# Cutoff Values of Noncycloplegic Spherical Equivalent Refraction for Myopia Detection in Southwestern Chinese Children

**DOI:** 10.1155/joph/9984560

**Published:** 2025-12-23

**Authors:** Cong Zhang, Xue Qing Zhao, Yue Yan, Jingying Wang

**Affiliations:** ^1^ Shapingba Hospital Affiliated to Chongqing University (Shapingba District People’s Hospital of Chongqing), Chongqing, 401331, China; ^2^ Department of Pediatrics, Chongqing Hospital of Traditional Chinese Medicine, Chongqing, 400021, China; ^3^ Department of Medical Technology, Chongqing Medical and Pharmaceutical College, Chongqing, 401331, China, cqmu.edu.cn

**Keywords:** axial length, corneal radius of curvature, cross-sectional study, myopia detection, spherical equivalent refraction

## Abstract

**Objectives:**

Cycloplegic refraction, the gold standard for detecting myopia, is not available to everyone, even in hospital‐based scenarios. Nevertheless, the conclusions of previous studies investing in noncycloplegic indicators for myopia detection were inconsistent. We aimed to explore noncycloplegic indicators for detecting myopia in children from southwestern China.

**Methods:**

This retrospective cross‐sectional study collected data from 562 children aged 6–14 who visited the optometry clinic at Chongqing Shapingba District People’s Hospital in southwestern China between October 2023 and September 2024. The key noncycloplegic indicators, such as gender, age, uncorrected visual acuity (UCVA), intraocular pressure (IOP), average corneal curvature (ACC), noncycloplegic spherical equivalent refraction (NCSER), axial length (AL), AL to average corneal radius of curvature (AL/ACRC), height and weight, were estimated via logistic regression and receiver operating characteristic (ROC) curves to evaluate the efficacy of various indicators for detecting myopia (defined as cycloplegic spherical equivalent refraction ≤ −0.50 D).

**Results:**

A total of 562 subjects were included, with 72.2% diagnosed with myopia via cycloplegic refraction. Univariate logistic regression identified age, UCVA, NCSER, AL, and AL/ACRC ∗ 100 as predictors of myopia (CSER ≤ −0.50 D). The multiple logistic regression analysis revealed that UCVA, NCSER, and AL/ACRC ∗ 100 were significant indicators for myopia (CSER < −0.50 D). Although the area under the ROC curve (AUC) of the combined use of UCVA, NCSER, and AL/ACRC (AUC = 0.982) was the largest among the indicators under exploration, there was no significant difference from that of NCSER alone (AUC = 0.975) (*p* = 0.120). The cutoffs for NCSER were −0.815 D in the 6–8 year age range, −0.804 D in the 9–11 year age range, and −0.687 D in the 12–14 year age range.

**Conclusions:**

Our study of children (6–14 years) in southwestern China established NCSER as the most robust noncycloplegic indicator for myopia. Consequently, applying age‐specific NCSER thresholds may provide a practical clinical tool to guide decisions on both the necessity for cycloplegic refraction and the early implementation of myopia prevention strategies without initially requiring cycloplegia.

## 1. Introduction

Myopia is a refractive error resulting from an imbalance between the optical power of the refractive medium, mainly the cornea, and the axial length (AL) of the eye [1]. The prevalence of myopia among school‐aged children ranges from 54.71% to 75.35% [1]. In Guangzhou, the prevalence of myopia among 12 year olds rose from 49.7% in 2002 to 65.8% in 2014. Similarly, in Hong Kong, the prevalence of myopia among children younger than 6 years increased from 12.7% in 2015 to 25.2% in 2021, as diagnosed by cycloplegia [2]. In all, myopia is a prevalent and substantial problem in China. Blurred vision resulting from improperly focused retinal images can adversely affect children’s school performance. The children with uncorrected refractive errors had significantly lower average academic performance than those without refractive errors (mean difference = 21.55, *t* = 6.70, *p* < 0.001) [3]. Furthermore, compared to those without myopia, individuals with high myopia have significantly increased odds of developing myopic macular degeneration, retinal detachment, cataracts, and open‐angle glaucoma, by factors of 845.1, 12.6, 2.9, and 4.6‐fold, respectively [2]. The high prevalence and significant impact of myopia have made it a public health concern in China.

The prevailing common sense is that the earlier the onset of myopia occurs, the greater the likelihood of severe myopia. Over 50% of Chinese participants with myopia who develop myopia at ages 7 or 8 progress to high myopia in adulthood [4]. Notably, delaying the onset of myopia by 1 year reduces the risk of developing high myopia later in life. Thus, the Chinese government has implemented myopia screening strategies, including UCVA, noncycloplegic spherical equivalent refraction (SER) (NCSER), and even AL measurements, at least twice a year for earlier myopia detection and intervention.

Cycloplegic refraction has been established as the gold standard for detecting myopia. The younger the child, the more active the accommodation response; therefore, cycloplegic refraction is recommended [5]. However, many children have compliance problems because of uncomfortable feelings while using cycloplegic drugs. In addition, most parents hesitate to use cycloplegic refraction due to the inevitable side effects, including tearing, photophobia, transient loss of visual acuity, allergic conjunctivitis, potential corneal damage, and increased ocular pressure. Although rare, eye care providers should also consider several potential side effects of cycloplegia, including fever, tachycardia, convulsions, and delirium [6]. In all, cycloplegic eye drops are not available to everyone, even in clinics, let alone those used for screening as prescription drugs; one would only use cycloplegic medications if necessary to achieve cycloplegic SER (CSER) in practice.

Alternatively, noncycloplegic indicators such as UCVA [7–13], NCSER [5, 8, 10–12, 14, 15], AL [16–20], average corneal curvature (CC) (ACC) [16, 17], intraocular pressure (IOP) [21, 22], AL to average corneal radius of curvature (AL/ACRC) [5, 7, 12, 14, 15, 18–20], age [12, 14], gender [14], height [23–25], and weight [25, 26] are commonly used in myopia detection in both eye clinics and school screenings. Despite numerous studies examining the correlation between noncycloplegic indicators in children, the specific indicator for myopia detection remains inconsistent. Previous studies have recommended inconsistent approaches for myopia detection, proposing different cutoff values. These approaches include using AL/ACRC alone [19, 20], UCVA alone [9], the combination of UCVA and NCSER [7, 8, 10–13, 27], or the combination of AL/ACRC and NCSER [5, 14, 15]. This inconsistency obscures the most critical indicator(s) for effective myopia detection. Furthermore, existing evidence is primarily derived from cohorts in southeastern China (Supporting Table 1). Therefore, to identify optimal noncycloplegic indicators and their cutoff values for myopia detection specifically in children from southwestern China, we conducted this exploratory study.

## 2. Materials and Methods

The minimum sample size was calculated a priori using PASS software (Version 2021, NCSS, LLC) based on the precision of the areas under the receiver operating characteristic (ROC) curve (AUC). Assuming an expected AUC of 0.98 from a similar preliminary study [12] and targeting a 95% confidence interval width of 0.05, a minimum of 63 myopia cases (positive events) were required.

This retrospective cross‐sectional study included data from subjects aged 6–14 years who visited the optometry clinic at Chongqing Shapingba District People’s Hospital between October 2023 and September 2024. Children were included if they had an IOP of ≤ 22 mmHg, a best‐corrected visual acuity of 1.0 decimal (6/6) or better in each eye, were able to cooperate with all examinations, and had no ocular organic disorders. Participants were excluded for the following reasons: diagnosed systemic diseases (e.g., hypertension and autoimmune diseases); ocular conditions known to influence refractive error, such as allergic conjunctivitis, corneal opacity, amblyopia, glaucoma, fundus disease, strabismus, a history of using myopia management such as atropine, or a history of neurological or psychosomatic disorders, including convulsions or Tourette syndrome. Finally, 562 subjects aged 6–14 years, including 406 with myopia, were enrolled in this study, which received approval from the Human Studies Committee of Shapingba People’s Hospital, Chongqing. China (Approval No. KY202241), in accordance with the Declaration of Helsinki.

### 2.1. Ophthalmology Examination

All instruments were calibrated daily before the examination, and all operations were performed according to standard optometric routine procedures. The slit lamp examination (PS‐11E, Topcon. Japanese) and fundus examination (90D direct fundoscopy, Topcon. Japanese) were used to check for organic disorders. The monocular UCVA was assessed once in decimal notation at 5 m using a standard logarithmic visual acuity chart (XK100‐01, Xingkang Medical Tech, China). The IOP was measured three times via noncontact tonometry (FT‐1000, Tomey, Japan), and the average value was recorded. The NCSER was obtained using an autorefractor (KR‐1, Topcon, Japan), and biometric parameters including CC and AL were assessed with an IOLMaster 700 (Carl Zeiss Meditec, Germany). For autorefraction and biometric measurements, three measurements were taken. If the discrepancy between them exceeded 0.50 D for the spherical equivalent or 0.02 mm for AL, a fourth measurement was performed. The average of all valid measurements was used for the final analysis. Cycloplegia was induced by applying one drop of 1% cyclopentolate ophthalmic (Alcon, USA) for topical anesthesia, followed by another drop after a 5‐min interval. Pupil size and the light reflex were assessed 30 min after the final dose of the medication was administered. Cycloplegia was deemed adequate if the pupil size dilated to at least 6 mm; otherwise, a third drop was given. Finally, cycloplegic refraction was also assessed by autorefraction.

### 2.2. Statistical Analysis

In this study, the gold standard of myopia was defined as a CSER ≤ −0.50 D. In the data preprocessing phase, the SER was determined by adding half of the cylinder power to the sphere power, and the ACC was computed as the mean of the horizontal and vertical meridians of the corneal curvature. ACRC was calculated using the formula as follows: ACRC = 337.5/ACC, and the AL/ACRC ratio was obtained as the ratio of AL to ACRC. To mitigate the inflation of odds ratios (ORs), the ratio of AL to ACRC (AL/ACRC) was rescaled by a factor of 100 (AL/ACRC × 100) before being included in the logistic regression model. We selected the right eye for the final analysis because of the high correlation between the eyes.

Statistical analysis was conducted utilizing R software (Version 4.4.2; R Foundation for Statistical Computing, Vienna, Austria). A significance threshold of *p* < 0.05 was applied. The Shapiro–Wilk test was used to assess the normality of the quantitative variables. Variables were displayed as medians with interquartile ranges or proportions. Mann–Whitney *U* tests, or chi‐square tests, were used to compare the genders. The Wilcoxon signed‐rank test was used to compare the difference in SER before and after cycloplegia. The predictive performance of individual noncycloplegic indicators and their combination was evaluated for detecting myopia (defined as CSER ≤ −0.50 D). In the predictor selection process, variables that showed a significance level of *p* < 0.05 in univariate logistic regression and passed collinearity diagnostics were retained for further analysis using multiple logistic regression. The mean absolute error (MAE) and root mean square error (RMSE) show the average difference between the model’s predictions and the actual outcomes. Thus, we computed the MAE and RMSE based on the model’s predicted probabilities to quantify prediction performance. Furthermore, the predicted probabilities derived from the multiple regression models were used to generate ROC curves and to calculate the AUC.

## 3. Results

### 3.1. Demographic Characteristics

Of the 562 subjects who had complete data, 72.2% of the examined right eyes were diagnosed with myopia based on cycloplegic refraction. The observed high prevalence of myopia (72.2%) in our cohort is consistent with the study setting of a tertiary ophthalmology referral center, which may introduce a referral bias. The median NCSER was −1.50 D, the median CSER was −1.25 D, and there was a significant difference of 0.25 D in NCSER and CSER (*p* < 0.001, obtained from the Wilcoxon signed‐rank test). The ACC and AL were significantly different by gender (*p* < 0.05). Compared to boys, girls tended to have slightly steeper corneal curvatures (median: 43.1 vs. 43.6), shorter ALs (median: 24.4 vs. 23.8), and similar myopia magnitude (median: −1.25 D vs. median −1.25 D) (Table 1).

**Table 1:** Demographic characteristics of the cohort.

Parameters	Total, *N* = 562		Female (*N* = 285)	Male (*N* = 277)	*p* value
	Median (P25, P75)	Range	Median (P25, P75)	Median (P25, P75)	
Age (years)	10.0 (8.00; 12.0)	6–14	10.0 (8.00; 12.0)	10.0 (8.00; 12.0)	0.709
UCVA	0.30 (0.15; 0.60)	0.01–1.50	0.30 (0.15; 0.60)	0.40 (0.20; 0.60)	0.103
IOP (mmHg)	16.0 (14.0; 18.0)	9–22	16.0 (14.0; 18.0)	16.0 (14.0; 18.0)	0.606
ACC	43.4 (42.4; 44.2)	38.75–47.62	43.6 (42.7; 44.6)	43.1 (42.0; 44.0)	**< 0.001**
NCSER (D)	−1.50 (−2.50; −0.50)	−15.00–5.00	−1.50 (−2.75; −0.50)	−1.50 (−2.38; −0.63)	0.352
CSER (D)	−1.25 (−2.25; −0.25)	−15.00–6.13	−1.25 (−2.50; −0.25)	−1.25 (−2.13; −0.25)	0.503
NO. myopia (%)	406 (72.2%)		203 (71.2%)	203 (73.3%)	0.653
Height (cm)	145 (134; 157)	110–180	145 (133; 156)	145 (135; 159)	0.253
Weight (kg)	36.5 (28.0; 46.0)	17–95	36.0 (27.0; 45.0)	37.0 (30.0; 49.0)	0.086
AL (mm)	24.1 (23.4; 24.8)	19.86–27.51	23.8 (23.1; 24.5)	24.4 (23.6; 25.0)	**< 0.001**
AL/ACRC ∗ 100	309 (301; 317)	255.60–357.10	308 (300,317)	310 [304; 317]	0.074

*Note:* D, diopter. The bold *p* values (< 0.001) indicate that the differences between males and females for those specific parameters (ACC and AL) are statistically highly significant.

Abbreviations: ACC, average corneal curvature; AL, axial length; AL/ACRC, axial length to average corneal radius of curvature; CSER, cycloplegic spherical equivalent refraction; IOP, intraocular pressure; NCSER, noncycloplegic spherical equivalent refraction; UCVA, uncorrected visual acuity.

### 3.2. Correlation Analysis Between Noncycloplegic Indicators and Myopia (CSER ≤ −0.50 D)

The univariate logistic regression analysis revealed that age, UCVA, NCSER, AL, and AL/ACRC ∗ 100 were associated with myopia (CSER < −0.50 D). According to the principle of collinearity diagnosis [28]^,^ no collinearity among the variables was included in the multivariate logistic analysis (Supporting Table 2). We assessed variables with *p* < 0.05 in the univariate logistic regression via multiple logistic regression analysis. The multiple logistic regression model demonstrated excellent predictive accuracy, as indicated by a low MAE of 0.080 and a RMSE of 0.196. We found that NCSER, UCVA, and AL/ACRC ∗ 100 were significant indicators correlating to myopia (Table 2).

**Table 2:** Regression analysis between variables and myopia (CSER ≤ −0.50 D).

Characteristics	Univariate logistic regression analysis			Multiple logistic regression analysis		
	Coefficient estimate	OR (95% CI)	*p*	Coefficient estimate	OR (95% CI)	*p*
Intercept				−46.89	0	< 0.001
Gender	0.1	1.11 (0.77–1.61)	0.586			
Age (years)	0.37	1.45 (1.32–1.59)	**< 0.001**	0.1	1.11 (0.91–1.36)	0.322
UCVA	−7.59	0 (0–0)	**< 0.001**	−4.88	0.01 (0.00–0.06)	**< 0.001**
IOP (mmHg)	0.04	1.04 (0.97–1.12)	0.239			
ACC (D)	0.04	1.04 (0.91–1.19)	0.561			
NCSER (D)	−3.81	0.02 (0.01–0.04)	**< 0.001**	−2.16	0.12 (0.05–0.27)	**< 0.001**
AL (mm)	1.96	7.10 (5.08–10.32)	**< 0.001**	0.46	1.58 (0.86–2.89)	0.139
AL/ACRC ∗ 100	0.26	1.29 (1.24–1.36)	**< 0.001**	0.12	1.13 (1.05–1.21)	**< 0.001**

*Note:* D, diopter; 95% CI, 95% confidence interval. The bold values represent the factors that are strongly and consistently linked to myopia in this study, with UCVA, NCSER, and the AL/ACRC ratio emerging as key independent predictors.

Abbreviations: ACC, average corneal curvature; AL, axial length; AL/ACRC, axial length to average corneal radius of curvature; CSER, cycloplegic spherical equivalent refraction; IOP, intraocular pressure; NCSER, noncycloplegic spherical equivalent refraction; OR, odds ratio; UCVA, uncorrected visual acuity.

### 3.3. Effectiveness of NCSER, UCVA, AL/ACRC ∗ 100, and Their Combination for Myopia Detection

**Figure 1:**
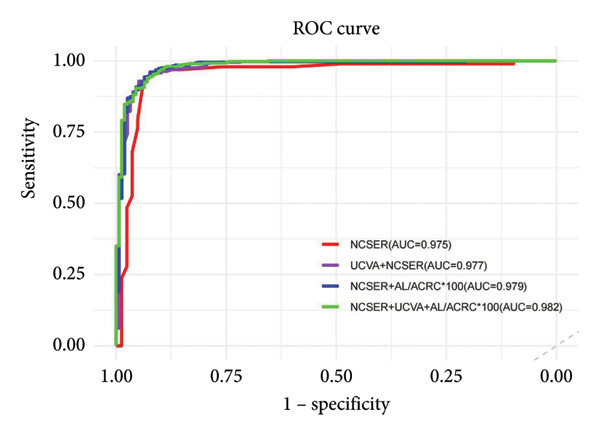
ROC curve of noncycloplegic indicators for myopia. ROC: Receiver operating characteristics; AUC: areas under the ROC curve; NCSER: noncycloplegic spherical equivalent refraction; UCVA: uncorrected visual acuity; AL/ACRC: axial length to average corneal radius of curvature.

Taking CSER ≤ 0.50 D as a reference standard, the combination of NCSER, UCVA, and AL/ACRC ∗ 100, yielding AUC values of 0.982, emerged as the most effective noncycloplegic indicator of myopia (Figure 1). However, this difference was not statistically significant compared to NCSER alone (*p* = 0.120). As shown in Table 3, NCSER demonstrated high diagnostic performance for myopia in Chinese children aged 6–14 years, with a sensitivity of 94.6%, a specificity of 93.6%, and a Youden index of 0.882, highlighting its practical utility. However, the optimal cutoff value for NCSER varied with age, as detailed in Table 4 (6–8 years: −0.815 D, 9–11 years: −0.804 D, and 12–14 years: −0.687 D).

**Table 3:** ROC analysis of NCSER, AL/ACRC, and UCVA for myopia assessment in children.

	Variables	AUC	95% CI	Cutoff value	Sensitivity	Specificity	Youden index
Myopia (CSER ≤ −0.50 D)	NCSER (D)	0.975	0.958 to 0.991	−0.813	0.946	0.936	0.882
	AL/ACRC ∗ 100	0.93	0.906 to 0.955	305.485	0.847	0.885	0.732
	UCVA	0.911	0.882 to 0.940	0.553	0.889	0.795	0.684
	UCVA + NCSER (D)	0.977	0.962 to 0.992	0.656	0.956	0.923	0.879
	UCVA + AL/ACRC ∗ 100	0.969	0.953 to 0.984	0.641	0.943	0.910	0.854
	NCSER + AL/ACRC ∗ 100	0.979	0.965 to 0.993	0.653	0.961	0.923	0.884
	NCSER + AL/ACRC ∗ 100 + UCVA	0.982	0.971 to 0.994	0.688	0.941	0.929	0.870

*Note:* D, diopter; AUC, area under the receiver operating characteristic curve; 95% CI, 95% confidence interval; AL/ACRC, axial length to average corneal radius of curvature.

Abbreviations: AL, axial length; NCSER, noncycloplegic spherical equivalent refraction; ROC, receiver operating characteristic; UCVA, uncorrected visual acuity.

**Table 4:** Cutoff, sensitivity, specificity, and Youden index of NCSER for myopia detection by group.

Age group (years)	No. total (myopia)	AUC	95% CI	Cutoff value	Sensitivity	Specificity	Youden
6–8	179 (97)	0.953	0.951 to 0.99	−0.815	0.959	0.915	0.873
9–11	198 (151)	0.992	0.981 to 1	−0.804	0.934	0.979	0.912
12–14	185 (158)	0.985	0.965 to 1	−0.687	0.987	0.926	0.913

*Note:* AUC, area under the receiver operating characteristic curve; 95% CI, 95% confidence interval.

Abbreviations: NCSER, noncycloplegic spherical equivalent refraction; ROC, receiver operating characteristic.

## 4. Discussion

This study establishes age‐specific cutoff values for noncycloplegic indicators, notably, NCSER, to identify myopia in children. Based on these cutoffs, children can be reliably classified as myopic, thereby helping clinicians decide when to initiate myopia prevention strategies or proceed with cycloplegic refraction for confirmation.

UCVA, AL, AL/ACRC, and NCSER, as well as their combinations, have been explored for myopia screening in previous studies [5, 7–10, 14, 27, 29, 30]. Some studies showed that AL/ACRC was the most important indicator for myopia detection [5, 7, 14, 30]. Some studies claimed that UCVA [9] or AL [29] was the indicator for myopia, and other studies recognized that NCSER was the leading indicator for myopia [8, 10, 27]. However, after adjusting for noncycloplegic indicators in the multiple logistic regression analysis, UCVA, NCSER, and AL/ACRC were identified as indicators for myopia (CSER < −0.50 D) in our study. The varying contributions of the indicators to CSER in different studies may be attributed to the differing preferences for noncycloplegic indicators across these studies (Supporting Table 1). In our study, ROC analysis further confirmed NCSER as the most effective noncycloplegic indicator for detecting myopia, outperforming AL/ACRC ∗ 100 and UCVA (Table 3). This finding aligned with previous studies, where the AUC of NCSER was reported to be 0.940 [8], 0.982 [12], and 0.93 [15], consistently surpassing the AUC values of UCVA, AL/ACRC, AL, and their combinations. Consistent with this interpretation, a prior study [27] reported only minimal improvement in myopic refraction error detection when combining age and UCVA with NCSER compared to NCSER alone (*R* ^2^ = 0.91 vs. *R* ^2^ = 0.90). Similarly, our analysis revealed that the combination of NCSER, AL/ACRC ∗ 100, and UCVA yielded marginally higher AUC values (0.982) than NCSER alone (0.975); however, this difference was not statistically significant (*p* = 0.167). These results collectively indicate that NCSER alone possesses sufficient predictive power for myopia detection, with limited clinical benefit gained through parameter combination in this specific population. Due to the similar efficacy of NCSER and the combined indicators, the lower expense of NCSER compared to the combined indicators in practice, NCSER, achieved through noncycloplegic autorefraction alone, was recommended as the optimal indicator for myopic children. However, there was a difference in NCSER and CSER (median: −1.50 D vs. median: −1.25 D), which aligned with prior research, indicating that noncycloplegic refraction yielded more myopic results than cycloplegic refraction [27, 31]. Thus, we should realize that, although there was no difference (*p* = 0.167) between NCSER (AUC = 0.975) and the combined use of NCSER, AL/ACRC ∗ 100, and UCVA (AUC = 0.982), NCSER by noncycloplegic autorefraction alone would increase the risk of misclassification of nonmyopic to myopic in practice.

This study established an NCSER cutoff of ≤ −0.813 D for myopia detection, yielding a sensitivity of 94.6% and a specificity of 93.6%. However, the cutoff was found to vary with age. This variability is consistent with the literature, which reported a cutoff of ≤ −0.75 D (sensitivity: 88.6% and specificity: 86.1%) in children aged 6–12 years [8] and a lower cutoff of ≤ −0.31 D (sensitivity: 97% and specificity: 83%) in a preschool cohort aged 3–6 years [15]. These discrepancies suggested that the age composition and geographic origin of the study cohort influence the diagnostic performance of NCSER. Otherwise, we found that the cutoff values of NCSER decreased as age increased (−0.815 D at 6–8 years; −0.804 D at 9–11 years; and −0.687 D at 12–14 years). The trend of less negative NCSER cutoffs with age likely reflects reduced accommodation in older children. Younger children have stronger accommodation ability, risking a false myopia reading of NCSER that necessitates a more negative cutoff to avoid overdiagnosis. In adolescents, diminished accommodation means that a more positive NCSER cutoff is more reliably indicative of myopia.

Some studies have reported that the ACC might be correlated with myopia development: a longitudinal study revealed a slight but significant (*p* < 0.001) flattening of the corneal curvature, and the onset of myopia was due to changes in the cornea, which could not compensate for the growth of the eyeball [17, 32]. However, we found that the ACC was independent of the degree of myopia. The difference may be due to our study’s cross‐sectional design, which did not follow individuals over time, or because our cohort’s age was above 4 years, and the cornea was more stable than it had been 4 years ago [33]. In addition, some studies have suggested that IOP is correlated with the magnitude [21]; however, we were unable to confirm this relationship through further multiple logistic regression analysis. The reason might be that the IOP in our study population was limited to the normal range.

The prevalence of myopia among children aged 6–14 years (72.20%) was lower than that reported in similar hospital‐based studies for those aged 8–18 years (87.67%) [20]. Due to the age being positively correlated with the amount of myopia [5, 34–36], the cohort with a low age range had a relatively low prevalence of myopia. However, we did not find a similar association between age and myopia in the multiple logistic regression analysis in our cohort, which may be because the age range (6–14 years) was not broad enough to detect.

Interestingly, there were gender differences in the ACC and AL; boys presented with flatter corneal curvatures (median: 43.1 vs. 43.6) and longer ALs (median: 24.4 vs. median 23.8) than girls did, which was also consistent with the large Chengdu cohort from southeastern China [17]. Theoretically, individuals with longer axial lengths are more likely to be myopic. Based on this inference, since boys generally have longer axial lengths than girls do, they should be more prone to myopia and have worse visual acuity. However, boys had a similar magnitude of myopia (median CSER: −1.25 D) to girls. The underlying reason may be that myopia progression differs according to gender, and myopia progression may involve mechanisms other than the elongation of AL relative to corneal curvature.

This study enabled us to observe noncycloplegic indicators for detecting myopia in children from southwest China. This study, to our knowledge, establishes the first hospital‐based cutoff values for the NCSER across various age groups. It comprehensively includes all standard noncycloplegic clinical indicators for myopia detection (Supporting Table 1). However, this study also has several limitations. First, as an exclusively hospital‐based cohort from southwestern China, the findings may lack generalizability to community‐based populations or other regions with different myopia prevalence rates. Second, the cross‐sectional design precludes the analysis of how predictive performance changes over time. Future longitudinal, multicenter studies with larger sample sizes are warranted to validate the role of these noncycloplegic indicators in detecting myopia.

## 5. Conclusion

We investigated the noncycloplegic predictors in a hospital‐based cohort of children aged 6–14 years from southwestern China. The NCSER, along with other variables such as AL/ACRC, was the most valuable noncycloplegic predictor for myopia. The cutoff values of the NCSER can be used in the eye clinic of southwestern China for further decisions on cycloplegic refraction and the adoption of myopia preventive measures.

## Ethics Statement

This study adhered to the principles outlined in the Declaration of Helsinki. The Medical Ethics Committee of the Chongqing Shapingba District People’s Hospital approved this study. The requirements for informed consent were waived due to the study’s retrospective nature and the minimal risks associated with it.

## Consent

Please see the Ethics Statement.

## Disclosure

All authors have approved the final manuscript.

## Conflicts of Interest

The authors declare no conflicts of interest.

## Author Contributions

Jingying Wang designed the study and wrote the manuscript. Cong Zhang collected the data and guaranteed its quality. Xue Qing Zhao analyzed the data. Yue Yan performed the relevant clinical measures.

## Funding

This work was supported by the Chongqing Municipal Education Commission under grant no. KJQN202202808 and the Health Commission of Chongqing Municipality under grant no. 2023MSXM050.

## Supporting Information

Supporting Table 1: Summary of studies reporting the myopia prediction model in different regions of China. Supporting Table 2: Collinear diagnosis of the variables (*p* < 0.05 in the univariate analysis).

## Supporting information


**Supporting Information** Additional supporting information can be found online in the Supporting Information section.

## Data Availability

The data supporting this study are available from the corresponding author upon reasonable request.
