# Supplementary material for: Cutoff Values of Noncycloplegic Spherical Equivalent Refraction for Myopia Detection in Southwestern Chinese Children
Source: J Ophthalmol. 2025 Dec 23;2025:9984560. doi: 10.1155/joph/9984560 (PMC12767027; doi:10.1155/joph/9984560)
Supplement: Supplementary file 1 — Supporting Information Additional supporting information can be found online in the Supporting Information section. [file JOPH-2025-9984560-s001.docx]

Supplement table 1. Summary of studies reporting the myopia prediction model in different region of China.

| Origin | study population | N | age range | Criteria | cutoff value of NSCER(D) | cutoff value of AL/ACRC | cutoff value of UCVA | cutoff value of AL (mm) | Conclusion |
| --- | --- | --- | --- | --- | --- | --- | --- | --- | --- |
| Peking in China(northeast) [5] | **hospital-based population** | 1024 | 4-6 | CSER≤-0.50D | not gained | 2.975 | not included | 23.235 | AL/ACRC was the better indicator for myopia than either AL or ACRC alone |
| Shanghai in China(southeast) [7] | school-based population | 4686 | 6-12 | CSER≤-0.50D | not included | 3.02 | 20/32 | 23.6 | The combination of AL/ ACRC and UCVA was found to be better myopia indicator than either AL/ ACRC or UCVA alone. |
| Shanghai in China(southeast) [8] | school-based population | 1687 | 6-12 | CSER≤-0.50D | -1.00D | not included | 20/32 | not included | Combination of UCVA and NCSER was better than either of the two tests alone for myopia screening |
| Shanghai in China(southeast) [9] | school-based population | 4416 | 6-12 | High myopia: CSER≤−3.0D | not included | not included | 20/50 | not included | Combination of distance vision acuity and near vision acuity test was more accurate for detecting myopia magnitude than either  of the two tests alone |
| Shanghai in China(southeast) [10] | school-based population | 6321 | 6-12 | CSER≤-0.50D | -0.75/-1.00 | not included | 20/30 | not included | The best screening measure of myopia was the combination of UCVA and NCSER. |
| Shanghai in China(southeast) [11] | school-based population | 2248 | 6-12 | CSER≤-0.50D | Not gained | not included | Not gained | not included | Combination of UCVA and NCSER produced the highest prediction performance for myopia  screening. |
| Shanghai in China(southeast) [12] | school-based population | 6017 | 4-15 | CSER≤-0.50D | Not gained | Not gained | 7-12 years:0.80 | not included | The combined use of UCVA and NCSER  tests and the combined use of AL/CR and NCSER tests achieved optimally  accuracy for myopia screening. |
| Zhejiang in China(southeast) [13] | school-based population | 3436 | 5-18 | CSER≤-0.50D | not included | 10-14 years old:3.06 | 10-14 years old:20/25 | 10-14 years old:24 | The AL/ACRC ratio alone or in combination with UCVA can be used as a tool for myopia screening. |
| Shanghai in China(southeast) [14] | school-based population | 7935 | 3-18 | CSER≤-0.50D | Not gained | 3.02 | not included | 23.71 | AL/ACRC was the best indicator for myopia but preschool children; AL/ACRC or AL combined with NCSER was recommended to myopia detection |
| Shanghai in China(southeast) [15] | school-based population | 1308 | 3-6 | CSER≤-0.50D | −0.31 | 2.93 | 0.35 | 22.86 | the  combination of AL/ ACRC and NCSER demonstrated favorable results for pre-myopia and  myopia screening of preschool children. |
| Nanjing in China(southeast) [18] | school-based population | 830 | 3-4 | CSER≤-0.50D | Not included | Not gained | Not included | Not gained | AL and AL/ ACRC could be used as obtainable indicators for identifying subjects at high risk of developing  premyopia and myopia in young preschool children |
| Hainan in China(southeast) [19] | **hospital-based population** | 187 | 18-35 | high myopia: CSER≤−6.00D | not included | 3.309 | not included | Not gained | AL/ ACRC can be used to diagnose high myopia in adults |
| Shenzhen in China(southeast) [20] | **hospital-based population** | 300 | 8-18 | CSER≤-0.50D | not included | 3.035 | not included | 23.63 | AL/ ACRC can be used to diagnosis of myopia |
| Shanghai in China(southeast) [27] | school-based population | 6825 | 4-15 | CSER≤-0.50D | not gained | not included | not gained | not included | Combination of NCSER, age and UCVA may help in identifying myopic magnitude |
| Shenzhen in China(southeast) [29] | school-based population | 1133 | 3-6 | CSER≤-0.50D | not gained | not gained | not included | not gained | For the SER variance, AL explained 18.6% and AL/ ACRC ratio explained 39.8% |
| Current study (Chongqing in China; **southwest**) | hospital-based population | 562 | 6-14 | CSER≤-0.50D | -0.813 | 3.05 | 0.553 | Not gained (excluded by multiple regression) | Our study of children (6–14 years) in southwestern China established non-cycloplegic spherical equivalent refraction (NCSER) as the most robust non-cycloplegic indicator for myopia. |

Not included: the study didn’t include the relative variable in the analysis.

Not gained: the study did analyze the relative variable without discussing the single cutoff value.

UCVA: uncorrected visual acuity, NCSER: non-cycloplegic spherical equivalent refraction, D: diopter, AL: axial length, AL/AC RC: axial length to average corneal radius of curvature. CSER: spherical equivalent refraction.

Supplement table 2. Collinear diagnosis of the variables (P<0.05 in univariate analysis)

|  | VIF | Tolerance |
| --- | --- | --- |
| Age(years) | 1.19 | 0.843 |
| UCVA | 1.15 | 0.869 |
| NCSER(D) | 1.08 | 0.925 |
| AL (mm) | 1.24 | 0.806 |
| AL/ACRC*100 | 1.3 | 0.771 |

UCVA: uncorrected visual acuity, NCSER: non-cycloplegic spherical equivalent refraction, D: diopter, AL: axial length, AL/ACRC: axial length to average corneal radius of curvature.
